# Bcl-2 protein expression is associated with p27 and p53 protein expressions and MIB-1 counts in breast cancer

**DOI:** 10.1186/1471-2407-6-187

**Published:** 2006-07-13

**Authors:** Shinichi Tsutsui, Kazuhiro Yasuda, Kosuke Suzuki, Hideya Takeuchi, Takashi Nishizaki, Hidefumi Higashi, Shoichi Era

**Affiliations:** 1 Department of Breast Surgery, Matsuyama Red Cross Hospital, Matsuyama, Japan; 2 Department of Surgery, Beppu Medical Center, Beppu, Japan; 3 Department of Surgery, Matsuyama Red Cross Hospital, Matsuyama, Japan; 4 Department of Pathology, Beppu Medical Center, Beppu, Japan

## Abstract

**Background:**

Recent experimental studies have shown that Bcl-2, which has been established as a key player in the control of apoptosis, plays a role in regulating the cell cycle and proliferation. The aim of this study was to investigate the relationship between Bcl-2 and p27 protein expression, p53 protein expression and the proliferation activity as defined by the MIB-1 counts. The prognostic implication of Bcl-2 protein expression in relation to p27 and p53 protein expressions and MIB-1 counts for breast cancer was also evaluated.

**Methods:**

The immunohistochemical expression of Bcl-2 protein was evaluated in a series of 249 invasive ductal carcinomas of the breast, in which p27 and p53 protein expressions and MIB-1 counts had been determined previously.

**Results:**

The Bcl-2 protein expression was found to be decreased in 105 (42%) cases. A decreased Bcl-2 protein expression was significantly correlated with a nuclear grade of III, a negative estrogen receptor, a decreased p27 protein expression, a positive p53 protein expression, positive MIB-1 counts and a positive HER2 protein expression. The incidence of a nuclear grade of III and positive MIB-1 counts increased as the number of abnormal findings of Bcl-2, p27 and p53 protein expressions increased. A univariate analysis indicated a decreased Bcl-2 protein expression to be significantly (p = 0.0089) associated with a worse disease free survival (DFS), while a multivariate analysis indicated the lymph node status and MIB-1 counts to be independently significant prognostic factors for the DFS.

**Conclusion:**

The Bcl-2 protein expression has a close correlation with p27 and p53 protein expressions and the proliferation activity determined by MIB-1 counts in invasive ductal carcinoma of the breast. The prognostic value of Bcl-2 as well as p27 and p53 protein expressions was dependent on the proliferation activity in breast cancer.

## Backgroud

The Bcl-2, first discovered in follicular and diffuse lymphomas possessing t(14;18) chromosomal translocation, has been established as a key regulator of apoptosis [1]. A decreased expression of Bcl-2 protein was shown to be associated with a poor clinical outcome in various human cancers including breast cancer [2-7]. Although Bcl-2 had been first thought to inhibit cell death without affecting cell proliferation, recent experimental studies have clarified the role of Bcl-2 in regulating the cell cycle and proliferation and that the Bcl-2 increased the level of p27 to regulate the cell cycle and progression [9-13]. Only a few studies, however, have investigated the relationship between Bcl-2 and p27 protein expressions in tumor specimens [14,15], while there have been many studies on the prognostic value of either Bcl-2 or p27 protein expression in various cancers [2-8,14,16-21]. The aim of this study, therefore, was to investigate the relationship between Bcl-2 and p27 protein expressions in breast cancer specimens, and the relationship between Bcl-2 protein expression and p53 protein expression, the proliferation activity as defined by the MIB-1 counts and HER2 protein expression was investigated. The prognostic implication of Bcl-2 protein expression in relation to p27 and p53 protein expressions and MIB-1 counts for breast cancer was also evaluated.

## Methods

### Patients

This study comprised 249 consecutive women with breast cancer who underwent surgery for breast cancer between 1985 and 1998 at the Beppu Medical Center Hospital, without any evidence of distant metastasis at the time of surgery. The histological type of breast cancer in all patients was invasive ductal carcinoma, while types other than invasive ductal carcinoma or non-invasive carcinoma were excluded in this study. The patients' ages ranged from 23 to 86 years, with a mean age of 58.1 years. The patients were treated either by a mastectomy (211 patients) or by breast conservation treatment (38 patients). Lymph node dissection was performed in 247 patients. Adjuvant postoperative hormone therapy was given to 220 patients and 215 patients received adjuvant postoperative chemotherapy, while 54 patients received postoperative radiotherapy. The median follow-up duration was 6.6 years. The institutional ethics committee granted ethical approval for the study. Although the informed consent was obtained for recent samples, it was not standard practice to obtain it for older samples.

### Immunohistochemistry

For the immunohistochemical analyses, 3-μm sections for Bcl-2 protein were dewaxed and rehydrated, and antigen retrieval was performed by microwave heating for 15 minutes in a 10 mM citrate buffer at pH 6.0. Next, the sections were reacted with mouse monoclonal antibody for Bcl-2 (Dako Japan, Kyoto, Japan) diluted at 1:100 for 60 minutes at room temperature, and then were subsequently stained using the universal immuno-peroxidase polymer method with a Histofine Simple Stain MAX PO(M) kit (Nichirei Corp., Tokyo, Japan) according to the protocol provided by the manufacturer. Positive reaction was visualized with diaminobenzidine, followed by counterstaining with hematoxylin. The sections from normal tonsillar tissue specimens were used as positive controls, while negative controls were performed by omitting the primary antibody.

The positivity of Bcl-2 protein expression was defined as having decreased when less than 25% of the tumor cells displayed a distinct cytoplasmic staining, since the cut-off values of 25% have been most frequently employed for Bcl-2 protein expressions [4,5]. The Bcl-2 protein expression was determined independently by three authors (S. T., K. Y. and S. E.) in whom two authors did not know any clinicopathological information for each patient. The methods and results of assessing p27, p53 and HER2 protein expressions and MIB-1 counts were described previously [22-24].

### Statistical analysis

The chi-squared test was used to investigate categorical variables. The disease free survival (DFS) was estimated using the Kaplan and Meier method, and any differences in the survival curves were compared by the Log-rank test. A multivariate analysis was performed by Cox's proportional hazards model. A *p *value of < 0.05 was regarded as statistically significant. All statistical analyses were performed using the StatView 5.0 software (SAS institute Inc., Cary, NC, USA).

## Results

**Figure 1:**
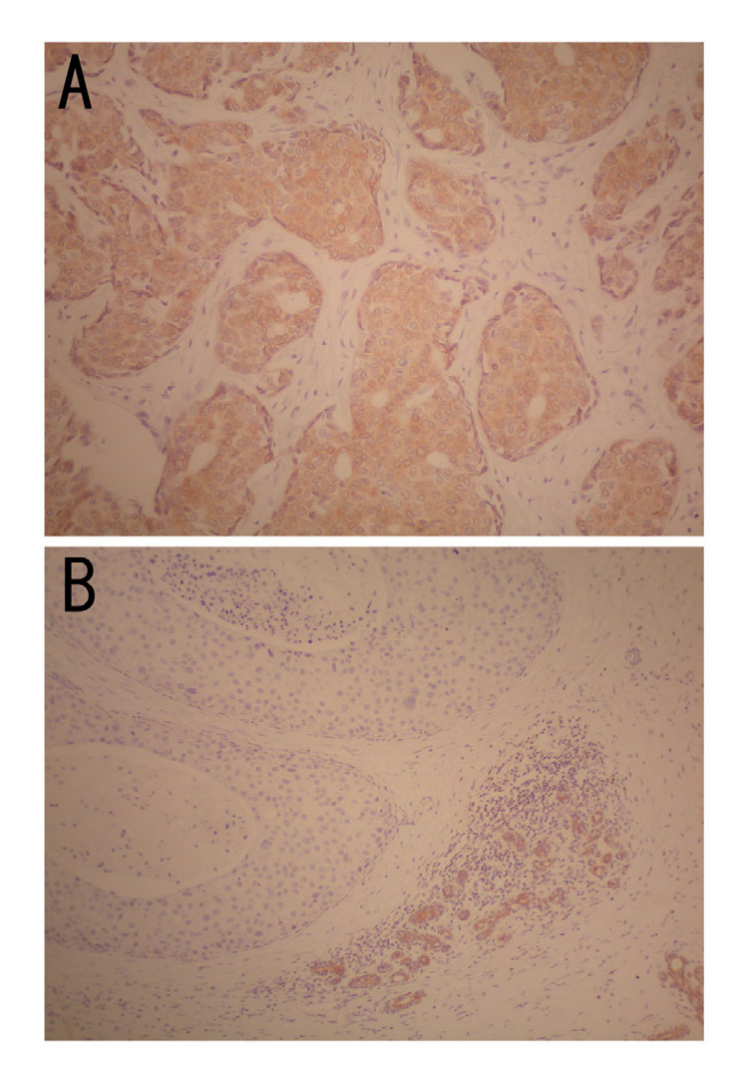
Immunohistochemical expression of Bcl-2 protein in breast cancer. A: a case with a normal expression of Bcl-2 protein in breast cancer cells. B: a case with a decreased expression of Bcl-2 protein, in which the expression of Bcl-2 protein of breast cancer cells decreased in comparison to a normal expression of Bcl-2 protein in normal mammary gland.

The Bcl-2 protein expression was determined to be normal in 144 (58 %) and decreased in 105 (42%) cases (Fig. 1). Table 1 shows the relationship between Bcl-2 protein expression and the clinicopathological factors including p27 and p53 protein expressions and MIB-1 counts in the breast cancers. The decreased expression of Bcl-2 protein significantly correlated with a nuclear grade of III, a negative estrogen receptor, a decreased p27 protein expression, a positive p53 protein expression and positive MIB-1 counts, while a decreased Bcl-2 protein expression was not significantly correlated with tumor size and lymph node metastasis. Table 2 shows the relationship between Bcl-2 and HER2 protein expression in 59 patients. The decreased expression of Bcl-2 protein significantly correlated with a positive expression of HER2 protein.

The positive expression of p53 protein and the decreased expression of p27 protein had been demonstrated to be associated with a nuclear grade of III and positive MIB-1 counts in our previous studies [22,23]. Table 3 shows the incidence of nuclear grade and MIB-1counts in relation to the combination of the abnormalities in Bcl-2, p27 and p53 protein expressions. The incidence of a nuclear grade of III and positive MIB-1 counts increased as the number of abnormal findings of Bcl-2, p27 and p53 protein expressions increased. There was a significant (p < 0.0001) correlation between the incidence of nuclear grade and MIB-1 counts and the number of abnormal findings of Bcl-2, p27 and p53 protein expressions.

**Figure 2:**
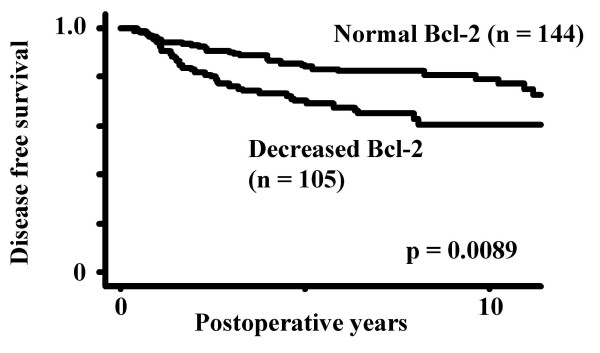
The disease free survival curves stratified by the Bcl-2 protein expressions in all 249 patients.

**Figure 3:**
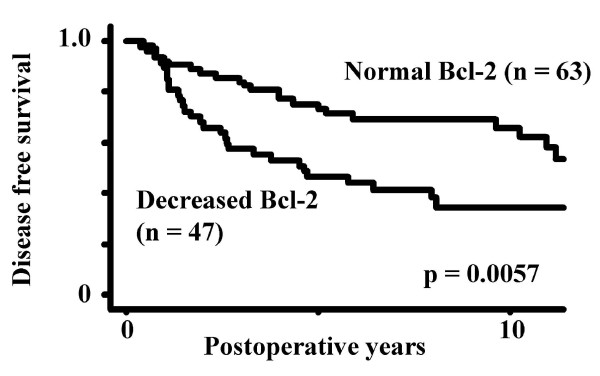
The disease free survival curves stratified by the Bcl-2 protein expressions in the 110 patients with lymph node metastases.

**Figure 4:**
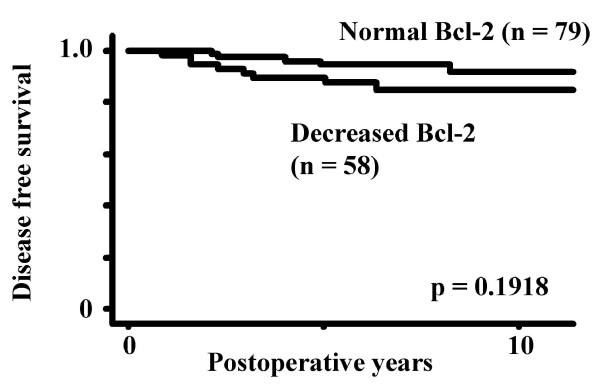
The disease free survival curves stratified by the Bcl-2 protein expressions in the 137 patients without lymph node metastases.

Univariate analyses for DFS indicated the patients with a decreased Bcl-2 protein expression demonstrated a significantly (p = 0.0089) worse DFS than those with a normal Bcl-2 protein expression (Fig. 2). The significant difference in the DFS was also demonstrated in the 110 patients with lymph nodes metastases (Fig. 3), while no significant difference in the DFS was found in 137 patients without lymph node metastases (Fig. 4). On the other hand, a multivariate analysis for the DFS indicated the lymph node status and MIB-1 counts to be independently significant prognostic factors for the DFS, while neither Bcl-2, p27 nor p53 protein expressions were independently significant prognostic factors for the DFS (Table 4).

## Discussion

The present study demonstrated a close correlation between Bcl-2 and p27 protein expressions regarding invasive ductal carcinoma of the breast. The decreased expression of Bcl-2 protein was significantly correlated to the decreased expression of p27 protein. Although there have been many studies on the prognostic value of each of Bcl-2 and p27 protein expressions in various human cancers [2-8,14,16-21], only a few studies have been conducted regarding the relationship between Bcl-2 and p27 protein expressions in the tumor specimens [14,15]. A significant correlation between Bcl-2 and p27 protein expressions was found in breast cancer specimens [14], while no correlation was found between the two protein expressions in oral and oropharygeal cancers [15].

Although the role of Bcl-2 has been established as a key player in the control of apoptosis, recent experimental studies have demonstrated Bcl-2 to be implicated in regulating the cell cycle and proliferation [9]. The Bcl-2 deficient T cells demonstrated an accelerated cell cycle progression [10]. On the other hand, the cells overexpressing the Bcl-2 gene product not only showed a delayed onset of apoptosis but also a rapid arrest in the G1 phase of the cell cycle [11], thus suggesting that Bcl-2 played a role in the transition from G0/G1 to S phase [9]. Futhermore, Linette et al. demonstrated that the increased level of Bcl-2 retarded the G0 to S transition, sustained the levels of p27 and repressed postactivation death [10]. Vairo et al. also demonstrated the Bcl-2 to retard the cell cycle entry by increasing the p27 and p130 levels [12]. Greider et al. demonstrated that Bcl-2 was unable to delay the S phase entry in p27 null cells, thus suggesting that p27 is required for the cell cycle regulation of Bcl-2 [13]. These experimental findings indicated that Bcl-2 directly increased the level of p27 [10,12,13], thereby regulating the cell cycle and progression [9].

The loss of the p53 function represents the most common genetic change known in human cancers. The present study demonstrated a significant correlation between p53 and Bcl-2 protein expressions in breast cancer. A significant inverse correlation was also demonstrated between the p53 and Bcl-2 protein expressions [2-5,10], while no correlation was found in other studies [9,14,18,20]. Haldar et al. showed that the overexpression of mutant p53 in breast cancer (MCF-7) cell line induced the down-regulation of Bcl-2 at both the protein and mRNA levels [25]. On the other hand, the proliferation index is a potent biological marker which can be used to estimate the growth of tumors quantitatively and can aid in determining the prognosis. MIB-1 is expressed throughout the cell cycle in proliferating cells, but not in cells in either the G0 phase or the early G1 phase. The Bcl-2 protein expression was demonstrated to be associated with a high proliferative activity as measured by the S phase fraction in breast and colon cancers or the ^3^H thymidine labeling index in breast cancer [2,3,9].

The Bcl-2 protein expression correlated inversely with the HER2 expression. We previously found the ER status to correlate inversely with HER2 expression and that the patients with a positive HER2 expression had a worse prognosis than those with a negative HER2 expression [26], while a positive correlation was found between the Bcl-2 protein expression and ER status in the present series. A decrease in Bcl-2 protein expression is associated with a high HER2 expression. Freneaux et al. demonstrated that a higher histological grade, a higher mitotic index, a higher apoptotic index, and a lower rate of ER positivity were found in Brca1-associated breast cancers and that the rate of Bcl-2-positive tumors was lower in Brca1-associated cancers than in cancers without Brca1 mutation, thus suggesting that the decrease in Bcl-2 expression might account for the high apoptotic and proliferative rates observed in Brca1-associated breast cancers [27].

In the present study, multivariate analysis determined only the proliferative activity to be an independently significant prognostic factor. The prognostic significance of Bcl-2 and p27 protein expressions has been well demonstrated in various cancers [2-8,14,16-21]. Multivariate analyses determined the Bcl-2 protein expression to be an independently significant prognostic factor for breast cancer [4,6], while other studies, as well as the present study, determined Bcl-2 protein expression to not be an independently significant prognostic factor [2,3]. Whether the Bcl-2 protein expression is determined to be independently significant factor or not is due to the relationship between the Bcl-2 protein expression and the other variables included in the multivariate models. The prognostic value of the MIB-1 counts has been demonstrated to be the most powerful among the nuclear pleomorphim, p53 protein expression and MIB-1 counts in our previous study [23]. On the other hand, we previously demonstrated that DNA aneuploidy reflected the accumulation of the abnormalities in p53 protein expression, epidermal growth factor receptor, estrogen receptor and progesterone receptor, thus suggesting that DNA aneuploidy reflected the accumulation of the aggressiveness of these biological parameters [28]. In the present study, the incidence of the positive MIB-1 counts increased as the number of abnormalities regarding the Bcl-2, p27 and p53 protein expressions increased, thus suggesting that the proliferative activity determined by the MIB-1 counts reflected the accumulation of abnormalities in the Bcl-2, p27 and p53 protein expressions. This finding may account for the MIB-1 counts showing the most powerful prognostic value in the multivariate analysis.

## Conclusion

The present study demonstrated the Bcl-2 protein expression to be closely correlated with the p27 and p53 protein expressions and the proliferation activity determined by the MIB-1 counts in breast cancer. The incidence of positive MIB-1 counts increased as the number of abnormalities regarding the Bcl-2, p27 and p53 protein expressions increased, while only the proliferation activity was determined to be an independently significant prognostic factor based by multivariate analyses. These findings indicated that the prognostic implications of the Bcl-2, p27 and p53 protein expressions were dependent on the proliferation activity in breast cancer.

## Competing interests

The author(s) declare that they have no competing interests.

## Authors' contributions

ST conceived of the study, carried out the immunohistochemical analyses, participated in the design of the study and drafted the manuscript. KY carried out the immunohistochemical analyses. KS, HT, TN and HH participated in the design of the study and coordination. SE carried out the immnohistochemical analyses.

## Pre-publication history

The pre-publication history for this paper can be accessed here:



## Figures and Tables

**Table 1:** Relationship between the Bcl-2 protein expression and the clinicopathological factors in breast cancer.

	No. of patients	Bcl-2 protein expression (%)		
		
		normal	decreased	p value
Tumor size (cm)				0.0770
- 2.0	57	37 (65)	20 (35)	
2.1 – 5.0	162	95 (59)	67 (41)	
5.1 -	30	12 (40)	18 (60)	
				
Lymph node metastasis*				0.9507
absent	137	79 (58)	58 (42)	
present	110	63 (57)	47 (43)	
				
Nuclear grade				< 0.0001
I or II	168	122 (73)	46 (27)	
III	81	22 (27)	59 (73)	
				
Estrogen receptor (ER)				< 0.0001
positive	109	90 (83)	19 (17)	
negative	140	54 (39)	86 (61)	
				
p27 protein expression				< 0.0001
normal	140	111 (79)	29 (21)	
decreased	109	33 (30)	76 (70)	
				
p53 protein expression				< 0.0001
negative	183	123 (67)	60 (33)	
positive	66	21 (32)	45 (68)	
				
MIB-1 counts				< 0.0001
negative (< 10%)	147	101 (69)	46 (31)	
positive (≥ 10%)	102	43 (42)	59 (58)	

* Lymph node dissection was performed in 247 patients

**Table 2:** Relationship between the Bcl-2 and HER2 protein expressions in breast cancer.

	No. of patients	Bcl-2 protein expression (%)		p value
			
		normal	decreased	
HER2 protein expression				0.0203
0	28	18 (64)	10 (36)	
I	15	6 (40)	9 (60)	
II	5	0 (0)	5 (100)	
III	11	3 (27)	8 (73)	

* HER2 protein expression was analyzed in 59 patients

**Table 3:** Nuclear pleomorphism and MIB-1 counts in relation to the number of abnormal findings in Bcl-2, p27 and p53 protein expressions.

No. of abnormal findings in Bcl-2, p27 and p53 protein expressions*	No. of patients	Nuclear grade **		MIB-1 counts ***	
			
		I or II	III	negative	positive
no	97	92 (95)	5 (5)	76 (78)	21 (22)
one	59	38 (64)	21 (36)	35 (59)	24 (41)
two	58	28 (48)	30 (52)	24 (41)	34 (59)
all (three)	35	10 (29)	25 (71)	12 (34)	23 (66)

* Abnormal findings in Bcl-2 and p27 protein expressions were defined as a decreased expression, while an abnormal finding in p53 protein expression was defined as a positive expression.

** p < 0.0001, *** p < 0.0001

**Table 4:** Multivariate analyses for DFS.

Variables	p value	relative risk	95% CI
Tumor size (cm):			
2.1 – 5.0 (vs. – 2.0)	0.8905	1.06	0.49–2.26
5.1 – (vs. – 2.0)	0.2467	1.72	0.69–4.29
			
Lymph node metastasis:			
present (vs. absent)	<0.0001	6.14	3.30–11.4
			
Nuclear grade:			
III (vs. I or II)	0.2791	1.39	0.77–2.52
			
Estrogen receptor:			
negative (vs. positive)	0.8502	1.06	0.59–1.89
			
p27 protein expression:			
decreased (vs. normal)	0.8976	1.04	0.58–1.85
			
p53 protein expression:			
positive (vs. negative)	0.3988	1.29	0.71–2.34
			
MIB-1 counts:			
10%≤ (vs.<10%)	0.0029	2.29	1.33–3.96
			
Bcl-2 protein:			
decreased (vs. normal)	0.4037	1.34	0.68–2.64

## References

[B1] Kroemer G (1997). The proto-oncogene Bcl-2 and its role in regulating apoptosis. Nat Med.

[B2] Silvestrini P, Veneroni S, Daidone MG, Benini E, Boracchi P, Mezzetti M, DiFronzo G, Rilke F, Veronesi U (1994). The Bcl-2 protein: a prognostic indicator strongly related to p53 protein in lymph node-negative breast cancer patients. J Natl Cancer Inst.

[B3] Joensuu H, Pylkkanen L, Toikkanen S (1994). Bcl-2 protein expression and long-term survival in breast cancer. Am J Pathol.

[B4] Gasparini G, Barbareschi M, Doglioni C, Palma PD, Mauri FA, Boracchi P, Bevilacqua P, Caffo O, Morelli L, Verderio P, Pezzella F, Harris AL (1995). Expression of bcl-2 protein predicts efficacy of adjuvant treatments in operable node-positive breast cancer. Clin Cancer Res.

[B5] Barbareschi M, Caffo O, Veronese S, Leek RD, Fina P, Fox S, Bonzanini M, Girlando S, Morelli L, Eccher C, Pezzella F, Doglioni C, Palma PD, Harris A (1996). Bcl-2 and p53 expression in node-negative breast carcinoma: A study with long-term follow-up. Hum Pathol.

[B6] Le MG, Mathieu M, Douc-Rasy S, Le Bihan M, All HAE, Spielmann M, Riou G (1999). c-myc, p53 and bcl-2, apoptosis-related genes in infiltrating breast carcinomas: evidence of a link between bcl-2 protein over-expression and a lower risk of metastasis and death in operable patients. Int J Cancer.

[B7] Sinicrope FA, Hart J, Michelassi F, Lee JJ (1995). Prognostic value of bcl-2 oncoprotein expression in stage II colon carcinoma. Clin Cancer Res.

[B8] Chan W, Cheung K, Schorge JO, Huang L, Welch WR, Bell DA, Berkowitz RS, Mok SC (2000). Bcl-2 and p53 protein expression, apoptosis, and p53 mutation in human epithelial ovarian cancers. Am J Pathol.

[B9] Bonnefoy-Berard N, Aouacheria A, Verschelde C, Quemeneur L, Marcais A, Marvel J (2004). Control of proliferation of Bcl-2 family members. Biochim Biophys Acta.

[B10] Linette GP, Li Y, Roth K, Korsmeyer SJ (1996). Cross talk between cell death and cell cycle progression: BCL-2 regulates NFAT-mediated activation. Proc Natl Acad Sci USA.

[B11] Marvel J, Perkins GR, Rivas AL, Collins MKL (1994). Growth factor starvation of bcl-2 overexpressing murine bone marrow cells induced refractoriness to IL-3 stimulation of proliferation. Oncogene.

[B12] Vairo G, Soos TJ, Upton TM, Zalvide J, DeCaprio JA, Ewen ME, Koff A, Adams JM (2000). Bcl-2 retards cell cycle entry through p27^Kip1^, pRB relative p130, and altered E2F regulation. Mol Cell Biol.

[B13] Greider C, Chattopadhyay A, Parkhurst C, Yang E (2002). BCL-xL and BCL2 delay Myc-induced cell cycle entry through elevation of p27 and inhibition of G1 cyclin-dependent kinases. Oncogene.

[B14] Nohara T, Ryo T, Iwamoto S, Gon G, Tanigawa N (2001). Expression of cell-cycle regulator p27 is correlated to the prognosis and ER expression in breast carcinoma patients. Oncology.

[B15] Fujieda S, Inuzuka M, Tanaka N, Sunaga H, Fan G, Ito T, Sugimoto C, Tsuzuki H, Saito H (1999). Expression of p27 is associated with Bax expression and spontaneous apoptosis in oral and oropharyngeal carcinoma. Int J Cancer.

[B16] Porter PL, Malone KE, Heagerty PJ, Alexander GM, Gatti LA, Firpo EJ, Daling JR, Roberts JM (1997). Expression of cell-cycle regulators p27^Kip1 ^and cyclin E, alone and in combination, correlate with survival in young breast cancer patients. Nat Med.

[B17] Catzavelos C, Bhattacharya N, Ung YC, Wilson JA, Roncari L, Sandhu C, Shaw P, Yeger H, Morava-Protzner I, Kapusta L, Franssen E, Pritchrad KI, Slingerland JM (1997). Decreased levels of the cell-cycle inhibitor p27^Kip1 ^protein: Prognostic implications in primary breast cancer. Nat Med.

[B18] Lau R, Grimson R, Sansome C, Tornos C, Moll UM (2001). Low levels of cell cycle inhibitor p27kip1 combined with high levels of Ki-67 predict shortened disease-free survival in T1 and T2 invasive breast carcinomas. Int J Oncol.

[B19] Mori M, Mimori K, Shiraishi T, Ttanaka S, Ueo H, Sugimachi K, Akiyoshi T (1997). p27 expression and gastric carcinoma. Nat Med.

[B20] Sgambato A, Migaldi M, Leocata P, Ventura L, Criscuolo M, Di Giacomo C, Capelli G, Cittadini A, De Gaetani C (2000). Loss of p27^Kip1 ^expression is a strong independent prognostic factor of reduced survival in N0 gastric carcinomas. Cancer.

[B21] Loda M, Cukor B, Tam SW, Lavin P, Fiorentino M, Draetta GF, Jessup JM, Pagano M (1997). Increased proteasome-dependent degradation of the cylcin-dependent kinase inhibitor p27 in aggressive colorectal carcinomas. Nat Med.

[B22] Tsutsui S, Inoue H, Yasuda K, Suzuki K, Tahara K, Higashi H, Era S, Mori M (2005). The inactivation of PTEN is associated with a low p27Kip1 protein expression in breast cancer. Cancer.

[B23] Tsutsui S, Yasuda K, Higashi H, Tahara K, Sugita S, Eguchi H, Kayashima H, Miyazaki N, Muto Y, Era S (2004). Prognostic implication of p53 protein expression in relation to nuclear pleomorphism and the MIB-1 counts in breast cancer. Breast Cancer.

[B24] Emi Y, Kitamura K, Shikada Y, Kakeji Y, Takahashi I, Tsutsui S (2002). Metastatic breast cancer with HER2/neu-positive cells tends to have a morbid prognosis. Surgery.

[B25] Halder S, Negrini M, Monne M, Sabbioni S, Croce CM (1994). Down-regulation of bcl-2 in human breast cancer cells. Cancer Res.

[B26] Tsutsui S, Ohno S, Murakami S, Hachitanda Y, Oda S (2002). Prognostic value of c-erbB2 expression in breast cancer. J Surg Oncol.

[B27] Freneaux P, Stoppa-Lyonnet D, Mouret E, Kambouchner M, Nicolas A, Zafrani B, Vincent-Salomon A, Fourquet A, Magdelenat H, Sastre-Garau X (2000). Low expression of bcl-2 in Brca1-associated breast cancers. Br J Cancer.

[B28] Tsutsui S, Ohno S, Murakami S, Hachitanda Y, Oda S (2002). DNA aneuploidy in relation to the combination of analysis of estrogen receptor, progesterone receptor, p53 protein and epidermal growth factor receptor in 498 breast cancers. Oncology.

